# Supplementary material for: Iterative improvement in the automatic modular design of robot swarms
Source: PeerJ Comput Sci. 2020 Dec 7;6:e322. doi: 10.7717/peerj-cs.322 (PMC7924708; doi:10.7717/peerj-cs.322)
Supplement: Supplemental Information 3 [file peerj-cs-06-322-s003.zip › argos3/doc/api/standalone/a00440_source.html]

ARGoS: plugins/robots/foot-bot/control\_interface/ci\_footbot\_encoder\_sensor.cpp Source File


- Main Page
- Related Pages
- Namespaces
- Classes
- Files

- File List
- File Members

# plugins/robots/foot-bot/control\_interface/ci\_footbot\_encoder\_sensor.cpp

Go to the documentation of this file.

```
00001 
00007 #include "ci_footbot_encoder_sensor.h"
00008 
00009 #ifdef ARGOS_WITH_LUA
00010 #include <argos3/core/wrappers/lua/lua_utility.h>
00011 #endif
00012 
00013 namespace argos {
00014    
00015   /****************************************/
00016   /****************************************/
00017    
00018   const CCI_FootBotEncoderSensor::SReading& CCI_FootBotEncoderSensor::GetReading() const {
00019     return m_sReading;
00020   }
00021   
00022   /****************************************/
00023   /****************************************/
00024    
00025 #ifdef ARGOS_WITH_LUA
00026   void CCI_FootBotEncoderSensor::CreateLuaState(lua_State* pt_lua_state) {
00027     CLuaUtility::OpenRobotStateTable (pt_lua_state, "wheels"                                              );
00028     CLuaUtility::StartTable          (pt_lua_state, "encoder"                                             );
00029     CLuaUtility::AddToTable          (pt_lua_state, "distance_left",  m_sReading.CoveredDistanceLeftWheel );
00030     CLuaUtility::AddToTable          (pt_lua_state, "distance_right", m_sReading.CoveredDistanceRightWheel);
00031     CLuaUtility::AddToTable          (pt_lua_state, "axis_length",    m_sReading.WheelAxisLength          );
00032     CLuaUtility::EndTable            (pt_lua_state                                                        );
00033     CLuaUtility::CloseRobotStateTable(pt_lua_state                                                        );
00034   }
00035 #endif
00036 
00037   /****************************************/
00038   /****************************************/
00039 
00040 #ifdef ARGOS_WITH_LUA
00041   void CCI_FootBotEncoderSensor::ReadingsToLuaState(lua_State* pt_lua_state) {
00042     lua_getfield(pt_lua_state, -1, "wheels");
00043     lua_getfield(pt_lua_state, -1, "encoder");
00044     lua_pushnumber(pt_lua_state, m_sReading.CoveredDistanceLeftWheel);
00045     lua_setfield(pt_lua_state, -2, "distance_left");
00046     lua_pushnumber(pt_lua_state, m_sReading.CoveredDistanceRightWheel);
00047     lua_setfield(pt_lua_state, -2, "distance_right");
00048     lua_pop(pt_lua_state, 2);
00049   }
00050 #endif
00051 
00052 
00053   /****************************************/
00054   /****************************************/
00055 
00056 }
```

---

Generated on 10 Jul 2018 for ARGoS by 
 1.6.1
